# Area-Based Socio-Economic Inequalities in Mortality from Lung Cancer and Respiratory Diseases

**DOI:** 10.3390/ijerph16101791

**Published:** 2019-05-21

**Authors:** Maciej Polak, Agnieszka Genowska, Krystyna Szafraniec, Justyna Fryc, Jacek Jamiołkowski, Andrzej Pająk

**Affiliations:** 1 Department of Epidemiology and Population Studies, Jagiellonian University Medical College, 31-531 Krakow, Poland; krystyna.szafraniec@uj.edu.pl (K.S.); andrzej.pajak@uj.edu.pl (A.P.); 2 Department of Public Health, Medical University of Bialystok, 15-295 Bialystok, Poland; agnieszka.genowska@umb.edu.pl; 3 Department of Rheumatology and Internal Medicine, Medical University of Bialystok, 15-276 Bialystok, Poland; justyna.fryc@umb.edu.pl; 4 Department of Population Medicine and Civilization Diseases Prevention, Medical University of Bialystok, 15-269 Bialystok, Poland; jacek.jamiolkowski@umb.edu.pl

**Keywords:** area-based SES, respiratory diseases, lung function, ecological study

## Abstract

*Background*: After political transformation in 1989/1990, Poland experienced a general improvement in living conditions and quality of life, but the benefits did not extend evenly across all segments of the society. We hypothesized that the regional differences in mortality due to diseases of the respiratory system are related to socioeconomic status (SES) and its changes over time. *Materials and methods*: An ecological study was carried out in 66 sub-regions of Poland using the data from the period of 2010 to 2014. Age-standardized mortality rates (SMRs) were calculated separately for men and women in three age categories: ≥15, 25–64 years, and ≥65 years. An area-based SES index was derived from the characteristics of the sub-regions using the z-score method. Multiple weighted linear regression models were constructed to estimate a real socioeconomic gradient for mortality resulting from lung cancer and respiratory diseases. *Results*: In the regions studied, the SMRs for respiratory disease varied from 70/100,000 to 215/100,000 in men and from 18/100,000 to 53/100,000 in women. The SMRs for lung cancer varied from 36/100,000 to 110/100,000 among men and from 26/100,000 to 77/100,000 among women. After adjusting for the prevalence of smoking and environmental pollution, the SES index was found to be inversely associated with the SMR for lung cancer in each category of age among men, and in the age group of 25–64 years among women. An increase of the SES index between 2010 and 2014 was associated with a decrease of SMR for respiratory disease both in men and women, but this change was not significantly associated with the SMR for lung cancer. *Conclusion*: SES appears to be an important correlate of mortality from respiratory diseases and lung cancer at the population level, particularly in men. A lower SES was associated with greater mortality from lung cancer and respiratory diseases. An increase in SES over time was related to a decrease in mortality from respiratory disease, but not from lung cancer.

## 1. Introduction

Diseases of the respiratory system, such as chronic obstructive pulmonary disease (COPD), asthma, lower and upper respiratory tract infections, and lung cancer, generate high socioeconomic costs on a global scale. However, the burden of these diseases is unequally distributed around the world and in Europe [1,2]. Poland is among the countries showing high mortality rates in men. Poland’s mortality rate from respiratory diseases among men between 25–64 years is 30% higher than the rest of the European Union (EU). Nevertheless, in contrast to men, mortality rates for respiratory diseases are lower among women in Poland, as related to the EU average. In 2014, in the 25–64 age group, mortality from trachea, bronchus and lung cancer is higher in men and women in Poland compared to EU by 37% and 33%, respectively [3]. Diseases of the respiratory system constitute the fourth most common cause of death in Poland and the third most common cause of absence from work due to health problems, and they contribute to a significant burden in terms of costs related to loss of occupational productivity due to sick leave. In Poland in 2013, this corresponded to 2.3 billion Euro per year and 0.6% of the GDP [4]. COPD is one of the most common respiratory diseases among adults. According to the Polish part of the BOLD Study conducted in 2006, COPD was diagnosed in 22% of the population studied [5]. High prevalence of COPD is associated with increased expenses for treatment and rehabilitation (approximately 40 million Euro per year) and for the provision of social benefits (approximately 55 million Euro per year) [6].

Although smoking is considered to be the primary cause of COPD and lung cancer [7,8], other factors can also contribute to these conditions, including air pollution and work-related exposures [9,10]. In a study from Oslo, in 1992–1996, the relationship between air pollution and mortality due to COPD was found to be considerable for individuals over 50 years of age [11]. However, in recent decades, although tobacco consumption, air pollution, and work-related exposures decreased considerably in Poland [12,13], these changes were not followed by a significant decrease in mortality from lung cancer and COPD [14]. If the latter observation is not fully explained by the lag time between smoking or other exposures and the onset of COPD, it is likely that there are other driving forces, which contribute to morbidity and mortality resulting from the development of the two main respiratory diseases. In recent years, attention has been drawn to assess the association between low socioeconomic status (SES) and increased risk of chronic lung diseases. Indeed, based on studies from Norway, South Korea and different European cities, these diseases were found to be more common in deprived communities and in people with low levels of education [15,16,17]. Partially, these findings can be explained by differences in the prevalence of harmful health behaviors, including excessive alcohol consumption, smoking, and poor nutrition, as well as differences in access to healthcare. Attention has also been paid to the possible role of chronic psychosocial stress among this population [18,19].

Among the Central and Eastern European countries, Poland was the first country to initiate political changes and transition to a market economy in 1989 [20]. As a result, there was a gradual, general improvement in living conditions and quality of life, but the benefits did not extend evenly across all segments of the society [21]. Higher education contributed to better mobility at the labor market and eventually to higher income, while a reduction in the demand for manual work (the decline of heavy industry) led to a deterioration of the economic conditions of large groups of employees with a low level of education [21,22]. A poorer economic status of these groups was associated with the intensification of pre-existing social problems, including excessive alcohol consumption [21] and limited access to healthcare [23]. It is likely that growing social disparities could affect the regional diversification in morbidity and mortality due to the diseases of the respiratory system. 

We hypothesized that the regional differences in mortality due to diseases of the respiratory system are related to SES and its changes over time. The aim of this study was to assess the relationship between SES and its change from 2010 to 2014, assessed at the population level, and mortality due to respiratory diseases in 66 sub-regions of Poland.

## 2. Materials and Methods

### 2.1. Research Project

We conducted an ecological study, in which the observation units comprised 66 sub-regions of Poland, defined according to the geocoding standard Nomenclature des Unités Territoriales Statistiques (NUTS-3) of 2006 [24]. In general, the sub-regions have a similar number of residents (average = 583,786), with the exception of eight urban sub-regions, which are the largest cities in Poland, whose population varies from 410,252 to 1,700,112. The data for the analysis was obtained from Central Statistical Office (routinely collected data on a yearly basis), with the exception of the data on tobacco smoking, which was obtained from the Social Diagnosis research study [25]. 

### 2.2. Mortality

Mortality due to respiratory diseases (ICD-10: J00-J99) and malignant neoplasm of the trachea, bronchus, and lung (lung cancer) (ICD-10: C33-C34) in Poland between 2010 and 2014 was analyzed. The number of deaths in 5-year age groups, by gender and population size for the 66 sub-regions of Poland, formed the basis for the calculation of mortality rates, which were then standardized by a direct method to the Polish population size in 2010 [26]. 

The standardized mortality rates (SMRs) were analyzed by gender and three age groups, according to the official division of the working age population in Poland: ≥15 years (population of working age), 25–64 years (typical production age), and ≥65 years (common retirement age). The younger group (15–24 years) was not considered for analysis because very low mortality was observed in this group. Since there were fluctuations in mortality rates and no clear trend was found during the period from 2010 to 2014, the means from all five years were used in the analysis (mean SMRs).

### 2.3. Indicator of the Socioeconomic Status of the Sub-Region (Area-Based SES)

A synthetic SES index was calculated using the z-score method, based on the following partial indices for each sub-regions: Average monthly salary (PLN), people with higher education (%), people employed in finance and real estate (%), unemployment rate (%), and people on social support due to poverty (%). 

The partial SES indices were transformed to the level of stimulants (higher value of the indicator corresponds to a higher level of the studied phenomenon) and reduced to the standard normal distribution, which enables the addition of their values. Then, the average value of partial indicators was calculated. The synthetic index varies from −3 to 3, where values below zero characterize sub-regions with low SES, values close to zero indicate sub-regions with an average SES in Poland, and values above zero represent sub-regions with a higher than average SES [27]. The SES indicator for the sub-region was calculated for the years 2010 and 2014 and the change in this indicator was calculated by taking the difference of the values between 2014 and 2010.

### 2.4. Other Explanatory Variables

The quality of the natural environment in the sub-regions was evaluated based on the total emission of dust, sulfur dioxide and nitrogen oxides (tones/km^2^), industrial waste (thousands of tons/km^2^), and raw industrial sewage (dam^3^/km^2^). For separating the sub-regions with varying degrees of pollution, the method of cluster analysis of k-means was applied [28]. Moreover, the variables were log-transformed in order to obtain a symmetrical distribution. As a result, two clusters of the natural environment were distinguished, categorizing sub-regions into higher and lower pollution areas. 

The prevalence of cigarette smoking by sex group in sub-regions was calculated using the database of the nationwide Social Diagnosis study performed in 2011. The population density in sub-regions (the number of inhabitants per square kilometer) in the year 2010 indirectly determined the degree of urbanization of the sub-region. 

### 2.5. Statistical Analysis

The mortality rates in the sub-regions are presented as the mean values, according to age group and gender. The variability in mortality and other traits in the sub-regions between gender or age groups were compared using the relative variation coefficient (coefficient of variation, CV (%)).

A multiple linear regression model was used to assess the relationship between the SES of the sub-region and mortality due to respiratory diseases and lung cancer. Since the accuracy of the estimation of mortality rates depends on the size of the population, a weighted linear regression model was used, where the weight represented the size of the population in the sub-region. The results of models are presented as a beta coefficient with a 95% weighted confidence interval (95% WCI). Modeling included two scenarios:

1. The estimation of the relationship between mortality rate and the SES indicator of the sub-region calculated for the first year of observation (2010).
2. The estimation of the relationship between the mortality rate and the change in the SES indicator between the years 2010 and 2014.

Three linear models were constructed for each scenario (Model A, Model B, and Model C) for mortality due to respiratory diseases and mortality due to lung cancer. Model A estimated the relationship between the mortality rate and the SES indicator in 2010 (or in the second scenario with the change of the SES between 2010 and 2014). Additionally, the prevalence of tobacco smoking in the sub-region was included in Model B. Model C also takes into account the level of environmental pollution, by introducing the indicator variable (= 1) for sub-regions with high pollution, and the urbanization level of the sub-region. 

Statistical analysis was performed using the statistical software IBM® SPSS® Statistics for Windows, version 24.0 (IBM Corporation, Armonk, NY, USA). The level of statistical significance was α = 0.05.

## 3. Results

### 3.1. Mortality Due to Respiratory Diseases and Lung Cancer

Among men and women in the age group ≥15 years, the mean SMR was 72/100,000 and 46/100,000, respectively, during the period 2010–2014. The mortality due to respiratory diseases was characterized by high regional variability. The lowest mortality rates were observed in large cities (minimum in Krakow, 36/100,000 in men 25/100,000 in women), and rates three times higher were found in some non-urbanized sub-regions of north-central and north-eastern Poland. The largest regional variability was observed with regard to mortality due to respiratory diseases in women of age group ≥65 years (CV = 30%) (Table 1). Compared to the mortality due to respiratory diseases, the mortality from lung cancer was higher in men (SMR 101 vs 72/100,000) but lower in women (SMR: 36 vs 46/100,000). The mortality due to lung cancer was also characterized by similar regional variability. In men, the mortality rate varied from 70 to 215/100,000, while in women, it varied from 18 to 53/100,000 (Table 1).

### 3.2. Indicator of the Socioeconomic Status of the Sub-Region (Area-Based SES) and Environmental Pollution

**Figure 1:**
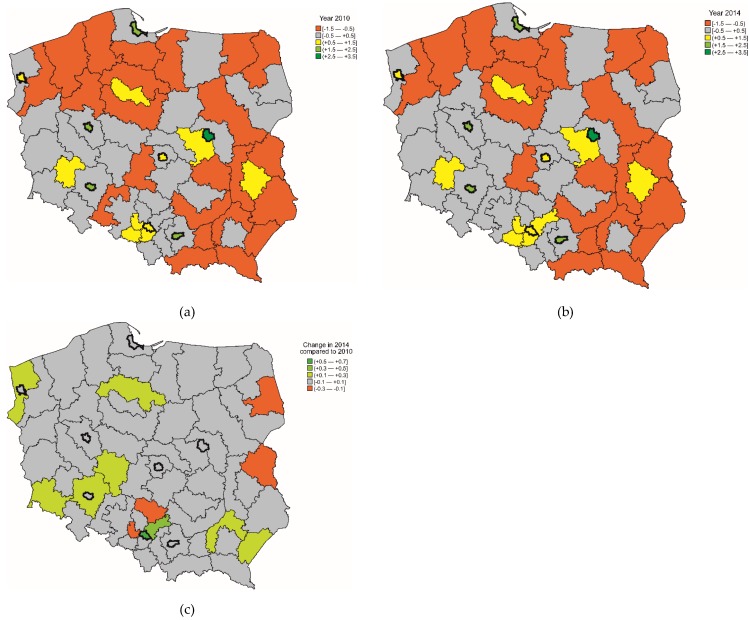
The socioeconomic status (SES) index in (**a**) 2010 and (**b**) 2014 and (**c**) its change.

In 2010, the synthetic SES index varied from −1.2 to 2.7. In 43 sub-regions (65%), the SES index values were below the average value; being highest in large cities and lowest in the regions of north and south-eastern Poland. In 2014, the synthetic SES index varied between -1.3 and 2.6. In 29 sub-regions (44%), the index was higher in 2014 compared to 2010 (Figure 1). 

The results of the cluster analysis revealed that 27 sub-regions (39%) showed higher environmental pollution. The percentage of smokers in the given sub-regions was higher in the male population (average value of 33.2%) compared to the female population (average value of 19.9%), and was characterized by greater regional variability among the female population (CV = 25%) when compared to the male population (CV = 16%). The population density in the analyzed sub-regions varied in the range of 44–3355 persons/km^2^ (median = 112 persons/km^2^).

### 3.3. Area-Based Socioeconomic Inequalities in Mortality

**Figure 2:**
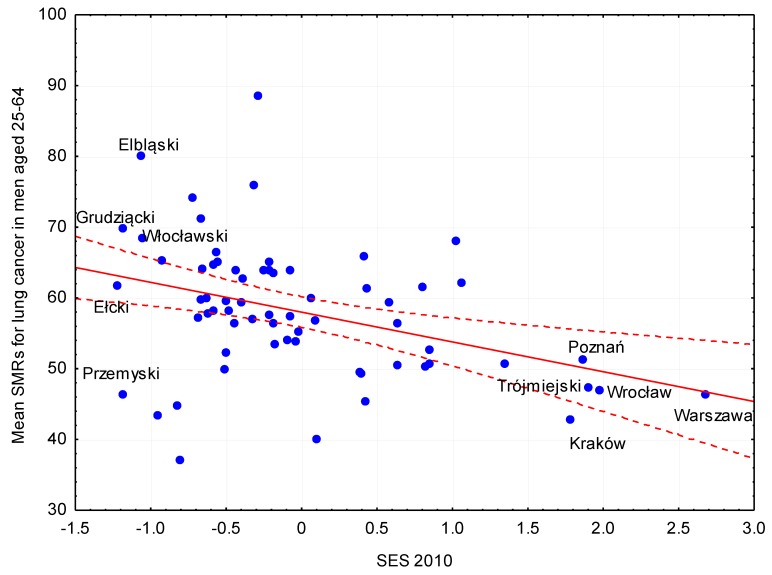
Relationship between SES index in 2010 and mortality due to lung cancer in men aged 20–64.

Table 2 shows the estimates of the relationship between the synthetic SES index of the sub-region and the mean SMR for respiratory diseases and lung cancer. In men, a higher SES was associated with lower mortality. In men aged ≥65 years, after adjusting for prevalence of tobacco smoking, an increase of the SES index by 1 standard deviation, resulted in a decrease of death rate by 41/100,000 for respiratory diseases and by 31/100,000 for lung cancer. Following adjustment for the quality of natural environment and degree of urbanization, the effect of estimated mortality (coefficient of regression) due to lung cancer reduced by 11%, but had no statistically significant effect on the relationship between SES and mortality due to lung cancer. The estimations in women were not found to be as consistent as in men. There was no significant association between SES and mortality due to respiratory diseases in any of the age groups. After adjusting for the prevalence of smoking and quality of the natural environment, a higher SES index was associated with lower mortality due to lung cancer only in the age group of 25–64 years (Figure 2). An improvement in the area-based SES index between 2010 and 2014 was significantly associated with lower mortality due to respiratory disease in both men and women aged ≥65 years. However, no significant association was observed between the change in the SES index and mortality due to lung cancer (Table 3). 

## 4. Discussion

### 4.1. Main Findings

In the present study, at the population level, we found a significant relationship between the SES, or its change, and mortality due to respiratory diseases and lung cancer among men. Among women, a statistically significant relationship was observed for mortality due to respiratory diseases in the age group of >15 years and for the change in SES and mortality from lung cancer in the age group of 25–64 years. An improvement in SES (an increase of the synthetic SES index) between the years 2010 and 2014 was associated with a significant reduction in mortality due to respiratory diseases among men and women, but no significant relationship was found with mortality due to lung cancer. 

The results of our study are consistent those reported by previous studies, which indicated higher mortality in men due to respiratory disease and lung cancer compared to women [14,16,29,30]. Our findings were also consistent with those reported by studies that were conducted outside of Poland on the relationship between the deprivation index and mortality due to respiratory diseases [31,32] and lung cancer [16,33]. Quantitative comparisons with the results of other studies would be difficult due to the differences in the research methods used and addressing conclusions beyond the population studied is even harder. However, if the relationship between SES and respiratory diseases are observed in Poland, it seems likely that differences in SES could contribute to the explanation of regional differences in the incidence and mortality from respiratory diseases in the less homogenous countries and partially explain the differences between counties. Further, it could be expected that an improvement of pulmonary health follows a decrease of intra-population socioeconomic disparities and the increase of SES of the entire population.

In our study, change in SES at the population level was significantly associated with mortality due to respiratory diseases, but not with mortality due to lung cancer, a disease that is strongly related to smoking. It is likely that smoking rates did not change much within such a short time. This finding is in accordance with the results obtained in a study performed by Sánchez-Santos et al. [34]. However, it is possible that the association could be significant if the changes in SES are more dynamic or the observation period is longer than 5 years.

The lack of a significant relationship between area-based SES and mortality in women, which is consistent with other studies [30,31,32], may be explained by the fact that harmful health behavior is less frequent among women. For example, the frequency of smoking is low in women and they are less likely to undertake jobs that involve exposure to harmful conditions [35]. 

Our study did not address the problem of causality, but some unknown mechanism might contribute to the relationship between SES and diseases of the respiratory system. An important component of the synthetic SES index was higher education, which has a positive impact on improving one’s health through gaining a better knowledge of beneficial health behaviors, as well as the ability to communicate with the representatives of medical professions and follow their recommendations [36]. The reduction in mortality due to lung diseases is associated with the level of income, which determines the capacity to purchase medicines and subsequently taking preventive measures [17]. However, worse SES, observed in people burdened with poverty or unemployment, causes restrictions in access to medical care and health impairment through the development of psychosocial stress, leading to smoking or increased alcohol consumption [17,36]. However, these arguments cannot fully explain the relationship between SES and mortality due to lung cancer. 

The analyzed sub-regions are constituted by relatively larger administrative units. Therefore, the pollution rate does not accurately reflect the local exposure (the variation at the sub-region level). However, the inclusion of the measure of environmental pollution in the analysis resulted in a reduction of the area-based SES and hence, mortality. Some studies showed that environmental factors may not be related to mortality from respiratory diseases, in contrast to the findings reported by other studies [11,37,38].

### 4.2. Limitations and Strengths

The main limitation in the interpretation of our results concerns the ecological design of the study. The results obtained on the basis of aggregated data do not allow drawing definitive conclusions at the level of an individual entity and the problem of causality was not addressed. In addition, the precision of estimating the relationship in an ecological study depends on the number and the size of geographical units. The smaller the territorial unit, the less heterogeneity the population contains. In our study, the division into 66 sub-regions was done at a macro-level, i.e., the occurrence of heterogeneity of data in the sub-regions may cause an underestimation of the investigated relationship. In the adjusted models, we included the variables, which could confound the relationships investigated, by being related to mortality and unequally distributed by SES. The models did not include the available data on health service, as the variables provided by the state statistical system do not correlate with mortality from respiratory diseases and lung cancer (data not shown). However, some residual confounding is possible and the effect of the quality and access to health service could partially explain the relationship between SES and mortality from respiratory diseases, but less with mortality from lung cancer as the survival time after the first diagnosis is generally short [39,40]. A less important problem would be that in the analysis of the changes over time in mortality, we were able to use the data on smoking prevalence and on population density for one year only. It seems unlikely that substantial changes in population density or in smoking prevalence occurred within the span of five years.

Nevertheless, our study has several strengths. For the first time, the relationship between SES and its change, and mortality due to respiratory disease and lung cancer were examined. This took into account the environmental pollution index and the prevalence of smoking in 66 sub-regions of Poland, a country with relatively higher mortality rates from lung cancer and other respiratory diseases. It represents a region, which had undergone a rapid political and socioeconomic transformation in recent years. Further socioeconomic factors were found to have a strong relationship with health in the populations of Central and East Europe, including Poland [41,42,43]. Another advantage is the development of an area-based SES indicator. The assessment of a complex phenomenon, such as the socioeconomic situation by using only one characteristic (e.g., education), cannot produce reliable results. This applies to both individual and population-level studies [44]. The composite measure used in this study allowed for the assessment of the combined effects of five variables describing different dimensions of SES (i.e., it enabled capture of the multidimensional character of this phenomenon) [45]. A number of features and methods are available to construct an SES index model, but there is no single universal recommendation [46]. The parameters selected in our study are considered to be good indicators of deprivation in the developed countries. The SES index was calculated using the z-score method, which allowed for building an SES index model in other populations and assessing its relationship with health. We believe that we have provided information regarding macro-determinants of mortality from respiratory diseases, which might be useful for planning interventions at the regional level.

## 5. Conclusions

SES appears to be an important correlate of mortality from respiratory diseases and lung cancer at the population level, particularly in men. A lower SES was associated with greater mortality from lung cancer and respiratory diseases. An increase in SES over time was related to a decrease in mortality from respiratory disease, but not from lung cancer.

## Figures and Tables

**Table 1:** Descriptive statistics for mortality rates in 66 sub-regions of Poland during the period 2010–2014.

Age Group (Years)	Respiratory Diseases (J00-99)			Lung Cancer (C33-34)		
	Mean SMRs	Min–Max	CV (%)	Mean SMRs	Min–Max	CV (%)
men						
≥15	72.4	36–110	22	101	70–215	20
25–64	23.7	15–41	25	58.1	37–89	16
≥65	442.2	207–662	23	466.6	347–661	14
women						
≥15	46	26–77	22	36.3	18–53	25
25–64	9.6	5–16	25	26.7	14–41	24
≥65	207.7	100–357	30	103.9	51–173	29

Abbreviations: SMR, standardized mortality rate per 100,000 inhabitants; CV, coefficient of variation.

**Table 2:** Relationship between socioeconomic status (SES) in 2010 and mean SMR for respiratory diseases and lung cancer in 66 sub-regions of Poland.

Age Group (Years)	Model	Men						Women					
		Respiratory Diseases (J00−J99)			Lung Cancer (C33−34)			Respiratory Diseases (J00−J99)			Lung Cancer (C33−34)		
		β	95% WCI	*p*	β	95% WCI	*p*	β	95% WCI	*p*	β	95% WCI	*p*
**≥15 **	A	**−5.8**	(−10.23; −1.40)	**0.01**	**−7.4**	**(−13.06; −1.70)**	**0.012**	2.8	(−1.04; 6.55)	0.15	**3.3**	**(0.70; 5.90)**	**0.01**
	B	**−5.2**	**(−9.6; −0.79)**	**0.02**	**−6.8**	**(−12.58; −1.10)**	**0.02**	0.5	(−3.62; 4.68)	0.80	−0.4	(−2.60; 1.72)	0.69
	C	−2.0	(−6.63; 2.63)	0.39	−5.2	(−11.64; 1.19)	0.109	3.0	(−1.40; 7.39)	0.18	−0.1	**(−2.51; 2.29)**	0.93
**25–64 **	A	−0.6	(−2.34; 1.10)	0.47	**−4.2**	**(−6.8; −1.62)**	**0.002**	0.1	(−0.59; 0.84)	0.73	0.04	(−1.87; 1.96)	0.96
	B	−0.2	(−1.86; 1.42)	0.79	**−3.7**	**(−6.2; −1.17)**	**0.005**	−0.4	(−1.19; 0.32)	**0.25**	**−2.5**	**(−4.19; −0.86)**	**0.004**
	C	0.1	(−1.75; 1.93)	0.92	**−3.3**	**(−6.13; −0.47)**	**0.023**	−0.4	(−1.20; 0.47)	**0.39**	**−2.5**	**(−4.35; −0.63)**	**0.01**
**≥65 **	A	−42.9	(−71.71; −14.24)	0.004	−34.3	(−52.27; −16.36)	<0.001	14.1	(−4.26; 32.44)	0.13	9.6	(−2.34; 21.58)	0.21
	B	−40.4	(−69.44; −11.31)	0.007	−31.1	(−48.80; −13.49)	0.001	−6.6	(−29.80; 16.78)	0.68	−5.2	(−12.74; 1.18)	0.31
	C	−16.8	(−46.83; 13.15)	0.27	−27.7	(−47.53; −7.86)	0.007	−3.6	(−18.95; 11.75)	0.80	−3.7	(−11.26; 3.85)	0.49

Notes: Model A—crude coefficient; Model B—adjusted for prevalence of smoking; Model C—adjusted for prevalence of smoking, environmental pollution and population density. Abbreviation: SMRs, standardized mortality rate per 100,000 population. C33-C34 and J00-J99, ICD-10 codes (mortality from respiratory diseases and from malignant neoplasm of the trachea, bronchus, and lung cancer), respectively. Significant results in bold.

**Table 3:** Relationship between the change of socioeconomic status (SES) between 2010 and 2014 and mean SMR for respiratory diseases and lung cancer in 66 sub-regions of Poland.

Age Group (Years)	Model	Men						Women					
		Respiratory Diseases (J00−J99)			Lung Cancer (C33−34)			Respiratory Diseases (J00−J99)			Lung Cancer (C33−34)		
		β	95% WCI	*p*	β	95% WCI	*p*	β	95% WCI	*p*	β	95% WCI	*p*
**≥15**	A	**−29.7**	**(−58.96; −0.33)**	**0.048**	0.8	(−38.04; 39.67)	0.97	−20.4	(−44.96; 4.19)	0.10	12.1	(−5.42; 29.59)	0.17
	B	**−29.3**	**(−57.84; −0.71)**	**0.045**	1.2	(−37.32; 39.67)	0.92	**−30.5**	**(−53.94; −6.98)**	**0.012**	−0.7	(−13.59; 12.15)	0.91
	C	**−29.1**	**(−54.75; −3.39)**	**0.027**	1.3	(−36.26; 38.92)	0.94	**−31.5**	**(−54.1; −8.84)**	**0.007**	−0.9	(−13.82; 12.03)	0.89
**25–64**	A	−2.0	(−13.20; 9.26)	0.727	−4.3	(−22.48; 13.84)	0.64	0.6	(−4.04; 5.25)	0.79	9.4	(−2.8; 21.62)	0.13
	B	−1.8	(−12.29; 8.76)	0.738	−4.0	(−21.19; 13.17)	0.64	−1.1	(−5.62; 3.42)	0.63	2.5	(−8.14; 13.08)	0.64
	C	−1.8	(−12.31; 8.81)	0.742	−4.0	(−20.85; 12.95)	0.64	−1.2	(−5.70; 3.36)	0.61	2.2	(−8.35; 12.79)	0.68
**≥65**	A	**−226.6**	**(−418.05; −35.23)**	**0.021**	19.2	(−110.23; 148.55)	0.77	−109.1	(−227.59; 9.40)	0.07	31.4	(−27.02; 89.83)	0.29
	B	**−224.8**	**(−414.37; −35.32)**	**0.021**	21.2	(−102.83; 145.14)	0.73	**−156.0**	**(−269.96; −42.08)**	**0.008**	−12.0	(−54.24; 30.17)	0.57
	C	**−223.3**	**(−387.66; −58.98)**	**0.009**	21.7	(−98.96; 142.3)	0.72	**−161.1**	**(−270.55; −51.57)**	**0.005**	−12.2	(−54.75; 30.4)	0.57

Notes: Model A—crude coefficient; Model B—adjusted for prevalence of smoking; Model C—adjusted for prevalence of smoking, environmental pollution and population density. Abbreviation: SMRs, standardized mortality rate per 100,000 population. C33-C34 and J00-J99, ICD-10 codes (mortality from respiratory diseases and from malignant neoplasm of the trachea, bronchus, and lung cancer), respectively. Significant results in bold.
